# Spin Polarization Properties of Two Dimensional GaP_3_ Induced by 3d Transition-Metal Doping

**DOI:** 10.3390/mi12070743

**Published:** 2021-06-24

**Authors:** Huihui Wei, Jiatian Guo, Xiaobo Yuan, Junfeng Ren

**Affiliations:** School of Physics and Electronics, Shandong Normal University, Jinan 250014, China; 2018020535@stu.sdnu.edu.cn (H.W.); 2019020526@stu.sdnu.edu.cn (J.G.)

**Keywords:** spin polarization, transition metal doping, first-principles calculations

## Abstract

The electronic structure and spin polarization properties of monolayer GaP_3_ induced by transition metal (TM) doping were investigated through a first-principles calculation based on density functional theory. The calculation results show that all the doped systems perform spin polarization properties, and the Fe–doped system shows the greatest spin polarization property with the biggest magnetic moment. Based on the analysis from the projected density of states, it was found that the new spin electronic states originated from the p–d orbital couplings between TM atoms and GaP_3_ lead to spin polarization. The spin polarization results were verified by calculating the spin density distributions and the charge transfer. It is effective to introduce the spin polarization in monolayer GaP_3_ by doping TM atoms, and our work provides theoretical calculation supports for the applications of triphosphide in spintronics.

## 1. Introduction

Since the discovery of graphene in 2004, two-dimensional (2D) materials have become an emerging class of materials. Compared with bulk materials, 2D materials are highly attractive for nanoelectronics, nanophotonics and spintronics at the nanoscale [1,2,3,4,5,6]. However, graphene has the shortcomings of zero band gap, which makes it unable to control the transport of carriers effectively and limits its development in some electronic fields. Beyond graphene, a series of 2D materials were experimentally stripped from the bulk crystals [7]. These 2D materials have their own advantages and disadvantages, for instance, hexagonal h-BN possesses a structure similar to graphene, but its wide band gap makes it an insulator, resulting in low overall carrier mobility [8,9,10]. As one of the typical 2D materials, transition metal di-chalcogenides (TMDCs) have the natural advantage of direct band gaps, controlled carrier mobility and suitable semiconductor band gaps, but this material’s oxidation resistance is very weak and can easily change under oxygen-containing conditions [11,12,13,14,15]. Besides, there are many other 2D materials, such as phosphorene [16,17,18], phosphide [19,20], Mxenes (carbides and nitrides) [21,22,23], and so on.

Recently, a new type of 2D material family was found, i.e., monolayer triphosphide, which has the same hexagonal structure as black phosphorus and gives a prediction, which can be easily stripped off experimentally from corresponded layered bulk materials [24]. Moreover, up to now, there have been several kinds of triphosphide that were investigated, and most of them are based on theoretical simulations. The application prospects of triphosphide in the fields of water splitting, photocatalytic reactions, etc., were predicted [24,25,26,27,28,29]. For example, Jing et al. reported that monolayer GeP_3_ has an indirect band gap, high carrier mobility and strong interlayer quantum confinement [30]. Similar results are also predicted in BiP_3_ by Liu et al. [31]. GaP_3_, which is a triphosphide composed of the group-IIIA elements and P atoms, was predicted by Yao et al. They found that GaP_3_ is stable and has good light absorption characteristics in the ultraviolet and visible light regions and can provide promising catalysts for water splitting [32]. On the other hand, Sun et al. also reported that GaP_3_ and other triphosphides have low lattice thermal conductivity, high Seebeck coefficient, high carrier mobility and high-performance thermoelectric properties, which confirms that GaP_3_ can act as promising materials for applications in thermoelectricity and other energy fields [33].

Most 2D materials are nonmagnetic, and obtaining magnetism in 2D materials is the key to applying them to spintronics. Thus far, there are many ways to introduce magnetism into 2D materials, such as the introduction of dislocation [34], defects [35], 3d transition-metal (TM) atoms doping [36,37,38,39], surface adsorption [40,41,42,43], etc. [44,45]. More interestingly, monolayer triphosphide reports rarely in the field of spintronics. In this work, we chose monolayer GaP_3_ as one of the triphosphides to investigate their spin polarization properties. As mentioned above, monolayer GaP_3_ has many of advantages in the fields of water-splitting and thermoelectricity. However, there still a lack of studies of monolayer GaP_3_ in the research field of spintronics. Therefore, it is interesting to achieve spin polarization in GaP_3_ and enrich its applications in spintronics. In this article, we completely investigate the spin polarization properties of 2D monolayer GaP_3_ induced by 3d TM doping by means of first-principles calculation.

## 2. Materials and Methods

In this work, the plane wave method based on the density functional theory (DFT) was adopted for all the first-principle calculations as incorporated in the Vienna ab initio simulation package (VASP) code [46]. The projector augmented wave (PAW) method was performed for the description of electron–ion interactions of the system, and the plane wave cut-off energy was set to 500 eV [47,48]. The electron exchange-correlation function for describing the electron interactions is the generalized gradient approximation (GGA) with Pardew–Burke–Enserch (PBE) of parametrization [49]. It should be noticed that the PBE function underestimates the band gap of the system, but the tendency of band structure that adopts PBE function is unchanged. The Brillouin zone was sampled with a 5 × 5 × 1 Gamma-pack scheme grid during the structure optimization of the system. The vacuum layer of the z-direction was set to 20 Å for avoiding the influence of periodic boundary conditions. To completely relax all atoms in the supercell, the total energy and the convergence benchmark for the force were set to 10^−6^ eV and −0.001 eV, respectively. Firstly, the feasibility analysis of the doped system was given by calculating the bond length and the spin polarization energy. Secondly, we showed that the nonmagnetic intrinsic GaP_3_ obtains various magnetism after doped different 3d TM atoms. Then, we calculated and analyzed the electronic band structure in addition to the density of state (DOS) of various doped systems. Lastly, the spin density distributions for all doped systems were given.

**Figure 1:**
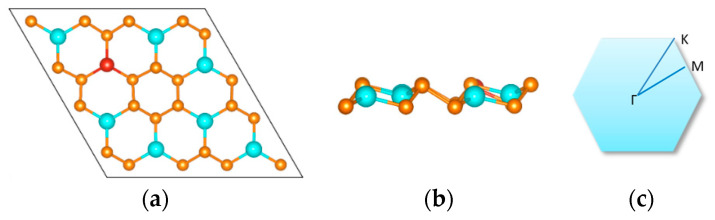
Top (**a**) and side (**b**) views of monolayer GaP_3_ structure. The blue, orange and red balls represent Ga, P and TM dopant (Ti, V, Cr, Mn, Fe, Co, Ni), respectively. The first Brillouin zone with high symmetry k points (Γ, M and K) is shown in (**c**).

To obtain a comprehensive understanding of monolayer GaP_3_, we first studied its structural properties. On the basis of our DFT calculations, as shown in Figure 1, the completely optimized doped structure is given, each dopant atom that connects to three P atoms forms three bonds, each P atom that connects one Ga atom and two P atoms forms two P–P bonds, and one Ga–P bond, respectively. There were 32 atoms in our supercell, and only one Ga atom was substituted by a 3d TM atom, so the dopant concentration was about 3.1%. The optimized lattice parameters of monolayer GaP_3_ are a = b = 7.21 Å, which is consistent with previous research [32].

## 3. Results

The bond lengths of various doped systems are shown in Table 1. It can be observed that the lengths of the P–P band and Ga–P band of the various doped system almost remain unchanged while the lengths of the dopant–P band change, but these such changes are subtle. It does not have much impact on the intrinsic structure, so these results show that the doped TM atoms can be stably embedded on the doped sites.

To gain deeper insight into the physics of these doped systems, we analyzed the spin polarization energy (E_pol_ = E_non_ − E_fer_), as shown in Table 1, which is defined as the energy difference between the nonmagnetic state (E_non_) and the ferromagnetic state (E_fer_). The positive value of E_pol_ means that the energy of the ferromagnetic state is lower than the energy of the nonmagnetic state. Hence, the doped system desires to become a ferromagnetic state. Our study shows that the GaP_3_ systems tend to be ferromagnetic states after doping TM atoms. In addition, we also calculated the magnetic moment of different doped systems, as shown in Table 1. The magnetic moment of pure GaP_3_ is 0 μB, this result indicates that the intrinsic structure is nonmagnetic, and when pure GaP_3_ obtains doped TM atoms, it obtains different magnetic moments with different TM atoms. Moreover, we found that the magnetic moment of the Fe–doped system is 5.00 μB, which is the biggest magnetic moment of various doped systems and means that the Fe–doped system can gain the strongest ferromagnetic coupling. On the other hand, the magnetic moment of the Ni–doped system is only 0.44 μB, which means that the Ni–doped system obtains the weakest ferromagnetic coupling corresponding to the lowest E_pol_. Therefore, it is feasible to obtain spin polarization in monolayer GaP_3_ by inducing TM atoms.

**Figure 2:**
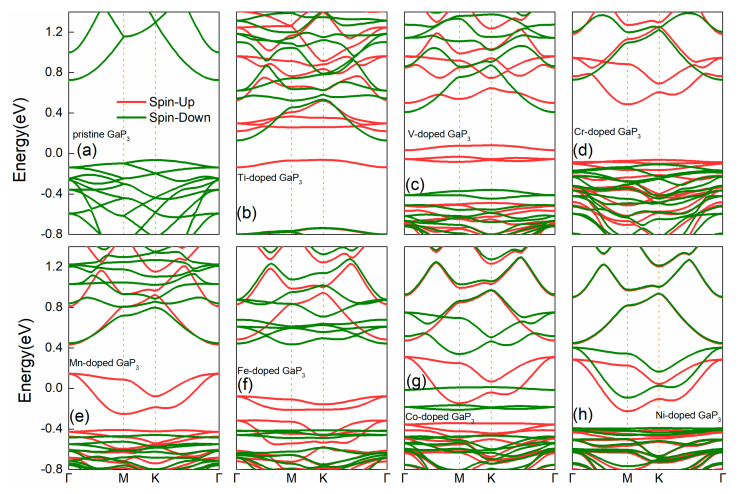
The electronic band structures of different doped GaP_3_ systems, (**a**) pure GaP_3_, (**b**) Ti–doped GaP_3_, (**c**) V–doped GaP_3_, (**d**) Cr–doped GaP_3_, (**e**) Mn–doped GaP_3_, (**f**) Fe–doped GaP_3_, (**g**) Co–doped GaP_3_ and (**h**) Ni–doped GaP_3_, respectively.

Figure 2 shows the band structures of various doped systems. It is clear that monolayer GaP_3_ is a semiconductor with an indirect band gap of 0.79 eV at the PBE level of theory. The conduction band minimum (CBM) locates at the Γ point, while the valence band maximum (VBM) locates at the K-point, which is consistent with previous research [32]. All bands are spin degenerate after considering electronics spin; this result demonstrates that monolayer GaP_3_ is totally no magnetism, agreeing well with the analysis above. When doping TM atoms, as shown in Figure 2b–h, the spin degeneracy is lifted, it can be seen that the band gap values are 0.87 eV, 0.77 eV, 0.55 eV and 0.84 eV, for the Ti–, V–, Cr–and Fe–doped systems, respectively. Moreover, for the Mn–, Co– and Ni–doped systems, the impurity levels induced by Mn, Co and Ni atoms pass through the Fermi level, which means that these three systems have semi-metallic properties. The whole systems show spin polarization properties apart from the pure structure. Through the calculations and the analysis from the magnetic moments, we can initially see that the Fe–doped system obtains the strongest spin polarization property, and the Ni–doped system shows the weakest spin polarization property.

**Figure 3:**
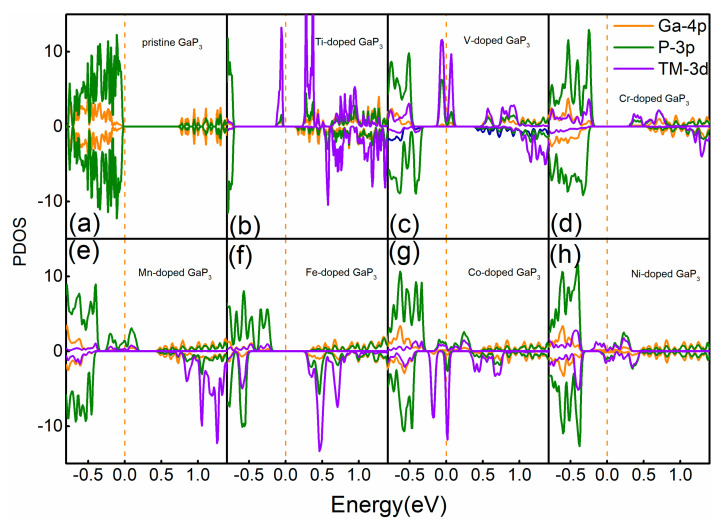
Projected density of states (PDOS) of the different doped systems, (**a**) pure system, (**b**) Ti–doped system, (**c**) V–doped system, (**d**) Cr–doped system, (**e**) Mn–doped system, (**f**) Fe–doped system, (**g**) Co–doped system and (**h**) Ni–doped system, respectively.

In order to investigate more about the spin polarization properties of various doped systems, we plotted the projected density of state (PDOS) near the Fermi level, as shown in Figure 3. It is clearly shown in Figure 3a that the pure GaP_3_ is spin energy degenerate for the spin up and spin down states, which means no spin polarization. Moreover, both the 4p orbital of the Ga atom and the 3p orbital of the P atom contributes the most to the electronic states of pure GaP3; in particular, the 4p orbital of the Ga atom plays the biggest part of it. Figure 3b–h shows the PDOS of various doped systems; we found that the spin-up and the spin-down electronic states split near the Fermi level, make the whole system asymmetric, and then the whole systems show the spin polarization properties. It is worth noting that the p–d orbital couplings between the TM atom and the GaP_3_ lead to the generation of the new spin states. Furthermore, the Fe– and the Ni–doped system possess the biggest and the weakest spin split, respectively, which means that the Fe–doped system has the greatest spin polarization and the Ni–doped system has the weakest spin polarization. These results correspond to the strongest and the weakest ferromagnetism, which agree well with the calculations of the magnetic moments in Table 1. Additionally, we also found an interesting result among the different doped systems. In the Fe– and Mn–doped systems, spin-up electronic states are mainly contributed by the 3p orbital of the P atom, while spin-down electronic states are mainly contributed by the 3d orbital of TM atoms near the Fermi level. However, in the Ti, V, Cr, Co and Ni–doped systems, both spin-up and spin-down electronic states are mainly contributed by the 3d orbital of TM atoms. The states near the Fermi energy are spin-nondegenerate, so the charges transferred from the TM to the GaP_3_ will fill these spin-polarized states, which make the TM doped GaP_3_ spin-polarized.

To further support the above, the charge transfers (Δq) of various doped systems were calculated by the Bader charge analysis [50], as shown in Table 1. In Table 1, the positive value means that the charge transfers from TM atoms to GaP_3_, vice versa. One can find that the values of Δq in the Ti–, V–, Cr–, Mn–, Fe–, Co– and Ni–doped systems are 1.22, 1.06, 0.86, 0.76, 0.60, 0.31 and 0.19 e, respectively. Different TM atoms in GaP_3_ show the different abilities to lose electrons. The Coulomb interaction between the different transfer charges of TM atoms and GaP_3_ causes the electronic structure to change differently, eventually leading to different spin polarization for various doped systems.

**Figure 4:**
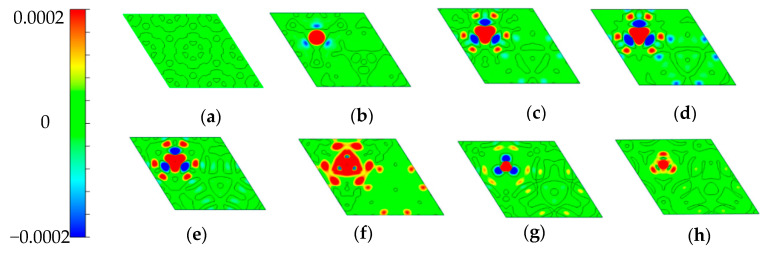
Spin density distributions for different doped systems. (**a**–**h**) correspond to pure GaP_3_, Ti–, V–, Cr–, Mn–, Fe–, Co– and Ni–doped, respectively. The 2D planes are determined by three Ga atoms and the dopants for the doped system, three P atoms for pure GaP_3_.

The spin density distributions of the various doped systems are also given in Figure 4. The spin density is defined as ∆ρs = ρ↑ − ρ↓, where ρ↑ represents the spin-up charge density, ρ↓ is the spin-down charge density. The red and blue regions in Figure 4 correspond to ∆ρs > 0 and ∆ρs < 0, respectively. It can be clearly seen that there is no spin density distribution in pure GaP_3_, as shown in Figure 4a, which means that the pure GaP_3_ has no spin polarization. However, we can find that the doped systems have spin density distributions but different from each other. Furthermore, the Fe–doped systems have the biggest area of spin density distribution, and then for the Mn–, Cr–, V–, Co–, Ti– and Ni–doped systems, the area of spin density contributions successively decrease. The whole results we obtained match well with the previous calculation of PDOS and magnetic moments. Therefore, introducing spin polarization properties by doping TM atoms is a good method for monolayer GaP_3_.

## 4. Conclusions

In our work, the electronic structure and the spin polarization properties of monolayer GaP_3_ induced by TM atoms (Ti, V, Cr, Mn, Fe, Co, and Ni) doping were investigated through the first-principles calculation based on density functional theory. The calculation of the bond lengths and the spin polarization energies in various doped systems were adopted to confirm the feasibility of doping TM atoms in monolayer GaP_3_. The various doped systems show different spin polarization properties, while the Fe–doped system can obtain the strongest magnetism than others. Based on the calculations of energy band electronic structures in TM–doped GaP_3_, it was found that elimination of spin degeneracy leads to asymmetry of the energy band, and then the band gaps in various doped systems changed. New spin electronic states originated from the p–d orbital couplings between TM atoms and GaP_3_ pass through the Fermi level, which leads to the semi-metallic property. Additionally, the spin density distributions and charge transfer for all doped systems also confirm that the TM atom doping can induce magnetism in GaP_3_. Our study provides a method for obtaining spin polarization in monolayer GaP_3_, which supports the excellent prospects for applying triphosphide in spintronic.

## Figures and Tables

**Table 1:** The bond length, spin polarization energy, charge transfer, and magnetic moment of various doped systems.

System		Ti-Doped	V-Doped	Cr-Doped	Mn-Doped	Fe-Doped	Co-Doped	Ni-Doped	Pure
Bond length (Å)	P-P	2.23	2.24	2.25	2.22	2.21	2.25	2.25	2.23
	Ga-P	2.36	2.35	2.35	2.37	2.36	2.34	2.35	2.36
	TM-P	2.42	2.37	2.38	2.32	2.23	2.23	2.24	-
E_pol_ (eV)		3.73	4.16	4.95	4.83	3.88	2.26	1.18	-
Δq (e)		1.22	1.06	0.86	0.76	0.60	0.31	0.19	-
Magnetic moment (μB)		1.00	2.00	3.00	4.00	5.00	1.44	0.44	0.00
